# Time to Discharge and its Predictors among Children Aged 1–60 Months with Severe Acute Malnutrition Admitted to the Therapeutic Feeding Center in A Tertiary Hospital, North West Ethiopia

**DOI:** 10.4314/ejhs.v32i6.7

**Published:** 2022-11

**Authors:** Mehretie Kokeb, Abinet Mariyo

**Affiliations:** 1 Department of Pediatrics and Child Health, College of Medicine and Health Sciences, University of Gondar, Ethiopia

**Keywords:** SAM, Time to discharge, Children, Gondar

## Abstract

**Background:**

Standard treatment of severe acute malnutrition with medical complication and/or failed appetite test is admission in therapeutic feeding centers for stabilization. Once stabilized, patients will be linked to Outpatient treatment program for rehabilitation. Information regarding time to discharge from inpatient therapeutic feeding centers is limited in Ethiopia. The main objective of this study was to assess the time to discharge and its predictors among children 1–60 months with Severe Acute Malnutrition admitted to University of Gondar Hospital.

**Methods:**

Hospital Based retrospective follow up study was conducted in Gondar University Hospital among 282 children aged 1–60 months admitted to inpatient Therapeutic Feeding Center from June 2018 to December 2020. Participants were selected by Simple random sampling technique. Time to discharge from inpatient treatment was estimated using Kaplan-Meir procedure and Log Rank test was used to test observed difference between covariates. Identification of predictors for time to discharge was done by Stratified cox regression model.

**Results:**

Overall 282 children were studied; 242 (85.8%) were discharged improved and 40 (14.2%) were censored. The median time to Discharge was 13 days (IQR: 9–18) and the Incidence of discharge was found to be 6.4 (95% CI: 5.6–7.2) per 100 person- day observations. Kwash-dermatosis (AHR=2.4, 95% CI: 1.17–4.8), Anemia (AHR=1.7, 95% CI: 1.1–2.6), pneumonia at admission (AHR=1.6, 95% CI: 1.01–2.63) and Hospital acquired infection (AHR=4.4, 95% CI: 2.4–8.2) were predictors of time to discharge.

**Conclusion:**

Hospital stay at the stabilization center was prolonged. Pneumonia, anemia, kwash dermatosis and Nosocomial infections were significant predictors of time to discharge.

## Introduction

Childhood malnutrition is one of the leading causes of morbidity and mortality in sub-Saharan Africa. Malnutrition refers to excess or deficient nutrient intake but in this study it is referred to as undernutrition. Under-nutrition manifests as wasting, stunting, underweight and evidence of micronutrient deficiencies. Severe Acute Malnutrition is defined by weight for height or length below -3-z-scores of the median according to World Health Organization growth reference (1).

Malnutrition predisposes children to different infections with a high rate of mortality, especially due to diarrheal diseases and lower respiratory tract infections (2). Malnutrition during infancy has a negative impact on brain growth and later intellectual development (3). Malnutrition contributes to a significant amount of morbidity and mortality in young children, and globally, malnutrition is a contributor to 52.5% of all deaths in young children (4). Sub-Saharan African countries are the most affected regions by malnutrition. A meta-analysis of the prevalence of malnutrition in 32 Sub-Saharan African countries based on cross-sectional data from Demographic Health Surveys from 2006 to 2016 found that wasting was prevalent in West Africa, with 18% in Niger, 15.5% in Burkina Faso, and 12.7% in Mali, respectively. Among East African countries, the prevalence was 11.1% and 8.7% in Comoros and Ethiopia respectively. One of the highest prevalence in Southern Africa was reported in Namibia (6.2%). In Central Africa, prevalence of wasting was 13% in Chad and 10.5% in Sao Tome (5). The most recent report from Ethiopia by the mini DHS report in 2019 reported the prevalence of wasting to be 7% (6).

Standard treatment of severe acute malnutrition with medical complications and/or a failed appetite test is admission in therapeutic feeding centers (stabilization Centers), stabilization with treatment of complications and nutritional support. (1). In general, the recommended length of stay at inpatient therapeutic feeding centers is 7–10 days, and for some children who can't tolerate ready-to-use therapeutic feeding, they will continue their treatment in the inpatient feeding center with the maximum recommended stay of 60 days (1). When compared to outpatient care, the expense of treating SAM in children at inpatient feeding programs is higher. Children with medical complications should be treated until recovered and discharged to OTP to reduce the health care cost and in hospital morbidity (7, 8). The length of stay in ITFC until stabilization among children with SAM is not well studied. Stabilization of a child with SAM is the initial step to rehabilitation and recovery (cure). Factors that affect stabilization directly affect time to recovery. We used predictors of recovery to conduct a literature search since there were few studies on predictors of stabilization. Discharge in this study refers to transfer to OTP after stabilization. The main objective of this study was to assess the time to discharge and its predictors among children aged 1–60 months of age with Severe Acute Malnutrition.

## Methods and Materials

**Study design and period**: An institution-based retrospective follow-up study was conducted among children aged 1 month to 60 months with SAM admitted to the Therapeutic feeding center in Gondar University Comprehensive specialized hospital from June 2018 to December 2020

**Study area**: A Hospital Based retrospective follow-up study was conducted in Gondar University Comprehensive Specialized Hospital (GUCSH) department of pediatrics and child health inpatient therapeutic feeding center. The Hospital is located in Gondar town 741km Northwest of the capital Addis Ababa. The hospital has different wards and there is a separate ward for children with SAM having 15 beds. Admission, Treatment, and discharge of patients with SAM is practiced according to the National Guideline for treatment of SAM 2019 (1)

**Source and study population**: The source population were all children age 1 –60 months with SAM admitted to GUCSH therapeutic feeding center and the study populations were children with SAM aged 1–60 months admitted to the TFC from June 2018 to December 2020.

**Inclusion and exclusion criteria**: All children aged 1–60 months with SAM admitted to the Therapeutic Feeding Center during the study period were included and Children with undiagnosed genetic disorders with SAM admitted to TFC, children having Down syndrome, cleft palate or any anatomic problems which interfere with feeding, children who had malnutrition secondary to cardiac illness, and neurosurgical conditions like Chiari malformations were excluded.

**Sample size and sampling procedure**: Sample size was calculated based on sample size estimation for survival analysis under cox proportional hazards assumption using Statistical software STATA version 14.0 taking the following assumptions (Table 1).

**Table 1:** Sample size determination

Assumptions	Predictors	AHR	Sample size
Power 90% α=0.05	Anemia	1.66	282
Withdrawal= 10% P event= 0.65	TB	2.03	144

The total sample size was 282 and the participants were recruited based on simple random sampling technique.

While time to discharge was dependent variable, age, sex, weight, length/height, MUAC, type of malnutrition, medical complications, comorbidity and routine medication were independent variables.

**The following operational definitions are used.**

**Died**: Dies while receiving treatment (1).

**Defaulted**: Absent for two consecutive days (1)

**Stabilized**: Condition has stabilized and referred to continue treatment in OTP (1).

**Discharge**: Patient has stabilized with no medical complication and able to continue treatment on OTP.

**Transfer out**: Moved to another facility for further medical care or moved out to receive care in another SC (1).

**Recovery**: reached the discharge criteria for SAM treatment (cured from malnutrition) (1).

**Length of Stay**: The number of days the child stayed in the hospital from admission until an event of interest occurred.

**Co-morbidity**: is a medical condition apart from SAM and its complication which was detected at admission or during hospital stay.

**Data collection procedure**: Data was collected from medical records by using a structured extraction form to get important information. The data extraction form was adopted from the Ethiopian SAM management protocol (1). Data was collected by a trained BSc nurse and Sociodemographic, anthropometric, medical issues, and medications given were gathered. Date of discharge or death and the outcome were taken from the discharge paper or death summary. The length of stay was computed for discharged children one by one using the difference between the date of admission to SC and the date of discharge from the hospital. Quality assurance of data was secured.

**Data processing and analysis**: Data were cleaned, coded and entered in to EpiData Version 3.1 after correct entry was checked and exported to STATA version 14 statistical software for analysis. Further data accuracy and missing values were checked on STATA. Descriptive analysis was reported using, graphs and frequency table. Time to discharge from ITFC was estimated using Kaplan-Meir procedure and then Log Rank test was used to test whether the observed difference between different groups of predictor variables was significant or not. Bivariate cox regression was done and variables with p value<0.2 were entered in to multivariate cox regression and p- value <0.05 was considered statistically significant.

AHR with 95% CI was used to show association. Schoenfeld residual analysis (global test) was used to show the Cox proportional hazard model assumption was not valid and stratified cox regression was done and proportional hazards assumption was valid with p value of 0.413. The Cox-Snell plot was used to check the overall model fitness.

**ETHICAL considerations**: Ethical clearance was obtained from Institutional Review Board of University of Gondar. The nature of the study didn't require informed consent from each client rather permission letter was obtained from Hospital administrator. The privacy of the participants were maintained by not including names and keeping the questionnaires locked.

## Results

**Socio-demography and anthropometry**: Among 282 children; 53.2% were males and the median age was 15 months (IQR: 2–59) with minimum and maximum age of 1 and 60months, respectively. Majorities (61.3%) of children were aged between 6 and 24 months and 11.3% were below 6months of age. Majorities (60.3 %) were severely stunted and 18.8% were moderately stunted. Microcephaly was identified in 4.26 % of children. Majority (66%) had marasmus and the rest were having kwashiorkor in 19.5% and had marasmic kwashiorkor in 14.5% (Table 2).

**Table 2:** Sociodemographic and Anthropometric Characteristics

Characteristics	Category	Frequency	Percent
**Age in months**	<6	32	11.3%
	6–24	173	61.3%
	24–60	77	27.3%
**Sex**	M	150	53.2%
	F	132	46.8%
**Length for age**	Normal	59	20.9%
	Moderate stunting	53	18.8%
	Severe Stunting	170	60.3%
**Head circumference**	Normal	268	95.%
	Microcephaly	12	4.3%
	Macrocephaly	2	0.7%
**Type of Malnutrition**	Marasmus	186	66%
	Kwashiorkor	55	19.5%
	Marasmic Kwashiorkor	41	14.5%

**Medical complication and comorbidity**: Out of the 282 children with SAM, 94.3% had medical complications at admission. Acute gastroenteritis was the commonest (48.2%) medical complication at admission followed by anemia (35.1%), dehydration (33.7%), pneumonia (33.3%), dermatosis (13.5%), sepsis (12.7%), Persistent diarrhea (12.4%), Shock (3.2%), meningitis (1.7%), hypoglycemia (1.4%) and the least was hypothermia (0.7%). Comorbidity was reported in 48.58% of children with SAM. The commonest comorbidity was Rickets which was detected in 29.% followed by Hospital Acquired infection (17.7%), Tuberculosis (3.5%), Malaria (3.2%), Vitamin A deficiency (2.8%), and pertussis (1.7%). HIV-infection was detected in 1.4% of children and Measles infection was diagnosed in 1% of children.

**Routine medicines and treatment**: Every child admitted with SAM received at least one routine medication. Among the routine medications; Ampicillin was given for majority (76.6%) of them followed by Gentamycin which was given for 72% of the children and Amoxicillin for 41.8% and deworming was given for 4.6%. Other antibiotics given were ceftriaxone (33.3 %,) cloxacillin (19.8%), vancomycin (12.7%), ceftazidime (12.4%), metronidazole (9.2%), ciprofloxacin (4.9%), Azithromycin (3.5%), Cotrimoxazole (1.4%) and Amikacin (0.3%). Considering micronutrient deficiency; Vitamin D stoss doss was given for 19.5%, high dose vitamin A for 3.5%, Folate for 4.6% and Zinc for 4.6% of children with SAM. Antimalarial drug was given for 3.2% and Anti TB was given for 2.8% of children with SAM. Antiretroviral and antifungal were given for 0.7% and 1.4% of children admitted with SAM, respectively.

**Figure 1:**
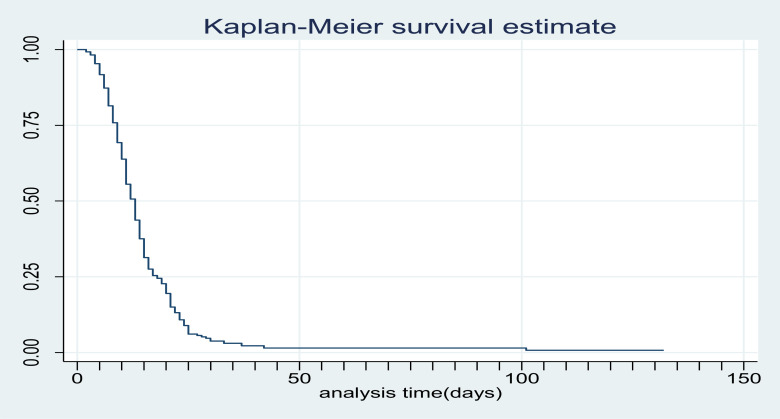
Overall survival estimates of children with SAM admitted to GUCSH during the study period

**Figure 2:**
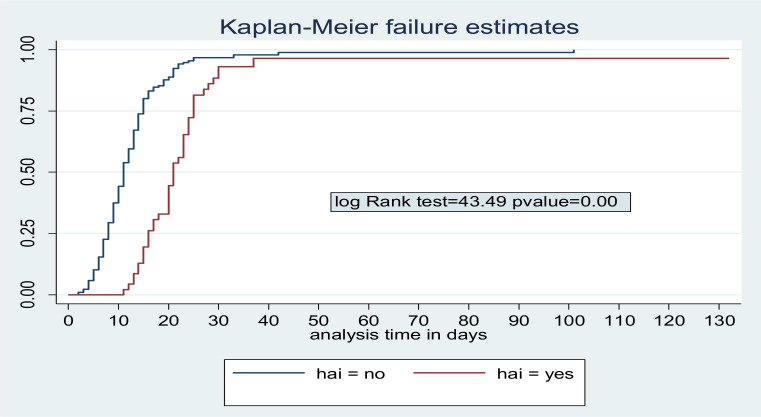
Log Rank failure estimate among children with SAM who had Hospital acquired infection (HAI).

**Figure 3:**
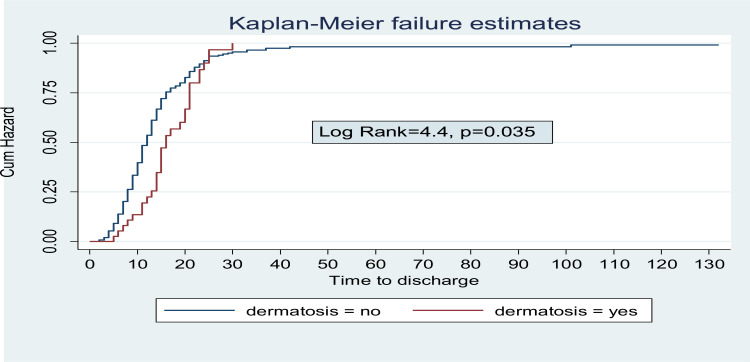
Log Rank failure estimate among children with SAM with dermatosis at admission.

**Figure 4:**
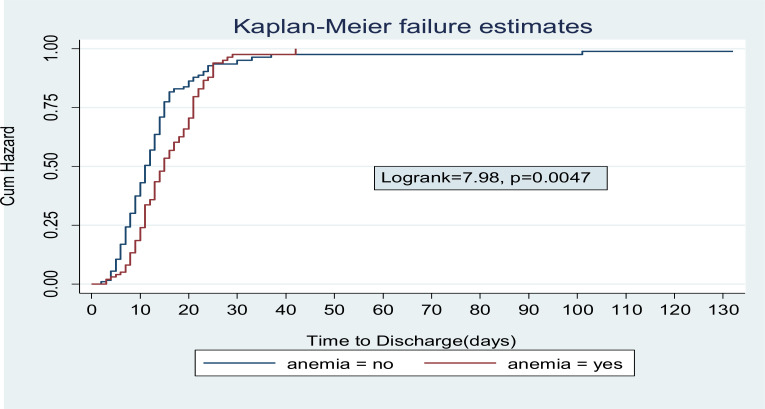
Log Rank estimate among children with SAM and Anemia.

**Treatment outcome and time of discharge from inpatient care for children with Severe Acute malnutrition**: Among 282 children admitted with SAM; 242 (85.8%) were discharged improved and 40 (14.2%) were censored (defaulted, died or transferred out). The median time to Discharge was 13 days (IQR: 9–18) (figure 1). Incidence of discharge was 6.4 (95% CI: 5.6–7.2) per 100 person- day observations. Majority (45.9%) of children were discharged in the 2^nd^ week of admission followed by 1^st^ week, 4^th^ week and 5^th^ week of discharge in 21.5%, 8.6% and 2.9%) of children, respectively. Children with SAM who had anemia, kwash dermatosis and hospital acquired infection were having longer Hospital stay compared with their counter parts. The median survival time for children with HAI, Kwash-dermatosis and Anemia was 21days (IQR: 16–35, 16 days (IQR: 13–21) and 15 days (IQR: 11–21), respectively (Figures 2–4).

**Predictors of time to Discharge from Inpatient treatment (stabilization center)**: By doing Bivariable cox regression analysis; Acute Gastroenteritis, Dermatosis, anemia, pneumonia, persistent diarrhea; rickets and Hospital acquired infection were found to be significant independent variables. Variables with p-value <0.2 in bivariable cox regression were analyzed by Stratified cox regression showing that Kwashdermatosis, anemia, pneumonia and Hospital acquired infection were predictors of time to discharge. Children without kwash-dermatosis at admission were 2.4(95% CI: 1.17–4.8) times to be discharged earlier compared with those who had kwash-dermatosis. Not having anemia at admission had 1.7(95% CI: 1.1–2.6) times higher probability of earlier discharge than those children who had anemia at admission. Children without pneumonia at admission had 1.6(95% CI: 1.01–2.63) times chance of being discharged than those who had pneumonia at admission. Those children who had no Hospital acquired infection were 4.4(95% CI: 2.4–8.2) times to be discharged earlier than those who didn't have hospital acquired infection.

## Discussion

This study was conducted to identify median length of stay and predictors for longer time to discharge in stabilization center at UOGCSH among children aged 1–60 months with severe acute malnutrition. In this study the rate of discharge after stabilization was found to be 85.8% while the remaining 40 (14.2%) were censored (defaulted, died or transferred out). Majority (72.7%) of the children with SAM admitted to the Stabilization center were below 24 months of age which is comparable with other studies done in the same region which is the age with greater risks of malnutrition (9,11,15). The predominant type of malnutrition identified during the study period was marasmus contributing for 66% which is comparable to other studies done in Axum, Bahirdar and Gondar (11,14,15) in contrary to the study done in Jima where most of the children admitted had edematous malnutrition (16). The most prevalent medical complication in this study was Acute gastroenteritis which is similar to the study done in Jimma (16) but in other studies it was not the major complication (9). The average length of stay in the stabilization center was 13 days which is longer than recommended stay at stabilization centers of 7 to 10 days (17, 18). This is because of the higher rate of HAI which made them to stay longer in the stabilization center to complete antibiotics. As to

In terms of predictors of time to discharge from inpatient treatment facility, Anemia, pneumonia, kwash dermatosis, and hospital acquired infections were significantly associated with a prolonged stay.

Children without kwash dermatosis were 2.4 (AHR=2.4, 95% CI: 1.17–4.8) times more likely to be discharged earlier than those with dermatosis which is in line with other studies (23, 24). The possible reason for the longer stay at inpatient feeding center for patients with kwash dermatosis may be related to the increased risk of infection due to the compromised skin integrity.

Those who didn't have anemia at admission were 2.6 times to be discharged earlier than those children with anemia. This is in line with a study conducted in Bahirdar which showed children who had no anemia at admission were 1.6times (AHR =1.552; 95%CI: 1.134, 2.124) discharged earlier than those with anemia at admission (11 This could be explained by the degree of reductive adaptation resulting in anemia may be due to prolonged nutrient deprivation which also affects other organ systems resulting longer time to recover.When compared to children with pneumonia at admission, those without it were 1.6 times more likely to be discharged early (AHR=1.5, 95% CI: 1.01–2.63) which is in line with a study done in Amahara region which showed Patients with pneumonia had a 34% lower rate of recovery when compared with those without pneumonia (AHR=0.66, 95% CI 0.53 to 0.83) (15). This could be due to the presence of infection resulting in increased metabolic demand which in turn increases nutrient loss and resulting in longer time to stabilization.

The presence of Hospital acquired infection significantly affected the time to discharge. Children with SAM who had no Hospital acquired infection were discharged 4.4(AHR=4.4, 95% CI: 2.4–8.2) times sooner than their counterparts. This is due to notorious organisms acquired at Hospitals resulting in need for potent antibiotics and longer duration of treatment. A study done in South African children admitted to hospitals reported that malnutrition, prolonged hospital stays and age less than 2 years were the major risk factors for nosocomial infections (25). In another study done among under-five children in Bangladesh, children with severe acute malnutrition had 2.5 (OR= 2.4, 95% CI: 1.1–5.2) times higher risk of acquiring Nosocomial infection (26). The prevalence of hospital acquired infection in our study was 17.7% which is similar with a prospective study done among 336 children with SAM in Jamaica which showed the prevalence of Nosocomial infection was 16%(27). Higher rate of Nosocomial infection among children with SAM could be explained by the depressed immunity in malnourished children which predisposes them to infection. To reduce in hospital morbidity and health care cost of children with SAM, strategies in the reduction of Hospital stay should be identified.

Despite our findings, the retrospective nature of the study prevented us from studying about the outcomes of some children due to undocumented discharge notes, forcing us to categorize them as censored cases, which had. an impact on the study's results and left some potential predictors out.

The strength of the evidence could have been stronger if this research had been conducted in multiple centers as opposed to one.

Our study concluded that the time to discharge after stabilization was longer than the recommended stay and children with Severe Acute Malnutrition who had medical complications like anemia; dermatosis and pneumonia had longer stay at stabilization center. The acquisition of Nosocomial infection among children with SAM prolonged their hospital stay. Therefore, infection prevention strategies should be implemented and we recommend further studies to be done in this area. the setting should devise mechanisms to reduce the length of stay of children with SAM at the stabilization center. Length of Hospital stay can be reduced by frequent checkup and care of children, avoiding overcrowding to reduce infection and do clinical audit to check the adherence of clinicians on management protocol and the setup is appropriate for malnourished children.
